# Analysis of disseminated tumor cells before and after platinum based chemotherapy in primary ovarian cancer. Do stem cell like cells predict prognosis?

**DOI:** 10.18632/oncotarget.8524

**Published:** 2016-04-01

**Authors:** Issam Chebouti, Christina Blassl, Pauline Wimberger, Hans Neubauer, Tanja Fehm, Rainer Kimmig, Sabine Kasimir-Bauer

**Affiliations:** ^1^ Department of Gynecology and Obstetrics, University Hospital Essen, Essen, Germany; ^2^ Department of Gynecology and Obstetrics, University Hospital Düsseldorf, Düsseldorf, Germany; ^3^ Department of Gynecology and Obstetrics, Carl-Gustav-Carus University, TU Dresden, Dresden, Germany

**Keywords:** disseminated tumor cells, bone marrow, stem cells, primary ovarian cancer

## Abstract

**Background:**

We recently reported that the presence of disseminated tumor cells (DTCs) in the bone marrow (BM) of primary ovarian cancer patients (POC pts) correlated with reduced progression free survival (PFS) and overall survival (OS). Here we analyzed whether the negative prognostic influence was related to DTC persistence after *platinum based chemotherapy* and/or due to DTCs associated with stem cell character.

**Results:**

DTCs were detected in 33/79 pts (42%) before and in 32/79 pts (41%) AT. Persistent DTCs were found in 13 pts, 20 pts were only positive BT, 19 pts AT and 27 pts had no DTCs. Whereas the presence of DTCs BT significantly correlated with reduced OS (*p* = 0.02), pts initially DTC_neg_ BT but DTC_pos_ AT had a significantly shorter PFS (*p* = 0.03). DTC persistence resulted in a shorter PFS and OS reaching borderline significance (*p* = 0.06; *p* = 0.07). LIN-28-and SOX-2 positive cells were detected in all eight pts AT.

**Patients and Methods:**

79 POC pts were studied for DTCs before therapy (BT) and after therapy (AT) using immunocytochemistry. Eight pts harboring at least five DTCs AT were further analyzed on two additional slides by four-fold immunofluorescence staining for DAPI, Cytokeratin (CK), SOX-2 or LIN-28, CD45 and CD34 (Cy5). A stem-like tumor cell was classified as Dapi_pos,_ CD45_neg,_ CD34_neg,_ SOX-2_pos_/LIN-28_pos_ and CK_pos_ or CK_neg._

**Conclusions:**

Stem cell associated proteins are expressed in DTCs that are present AT and their presence seem to be correlated with a worse outcome. Additional therapeutic regimens may be necessary to eliminate these cells.

## INTRODUCTION

Ovarian cancer is the fifth leading cause of all cancer related deaths in Europe and the United States and most tumors are diagnosed in an advanced stage with poor prognosis for the patients [1]. Conventional therapy is based on an initial debulking surgery aiming at macroscopic complete resection combined with subsequent platinum- and paclitaxel-based chemotherapy [2]. Postoperative residual tumor is one of the most important prognostic factors in advanced ovarian cancer [3, 4, 5].

It is hypothesized that cancer malignancy and metastasis are driven by a small subgroup of highly tumorigenic cells within the tumor, called metastasis initiating cells (MIC). These cells have the ability to self-renew, enhance tumorigenesis and are often found to be drug resistant [6]. The presence of such a small population, often referred to as cancer stem cells (CSC), has been confirmed in ovarian cancer cell lines as well as in tumor tissue [7, 8]. Their amount is increased in chemotherapy resistant ovarian cancer cell lines [7] and they are believed to contribute to an aggressive behavior of epithelial ovarian cancer [9]. The pluripotency associated stem cell factors SOX2 (sry related) and LIN-28 have been found to be expressed in ovarian cancer cell lines and tissue [10, 11, 12]. Bareiss et al., showed that SOX2 expression is a CSC marker in serous ovarian carcinomas (SOC) and can induce CSC properties [11]. In addition, SOX2 was reported to enhance migration and invasion of ovarian cancer cells [13]. Importantly, SOX2 overexpression was shown to be a poor prognostic marker in ovarian cancer [14] and also shown to be involved in taxane resistance [15, 16].

In ovarian cancer, the primary tumor usually metastasizes to the peritoneum, but a variety of studies including ours indicate that tumor cells frequently disseminate into the bone marrow (BM). Disseminated tumor cells (DTCs) in the BM are detected in 20% to 60% of cases before the onset of platinum-based chemotherapy depending on the method of detection used. Their prognostic relevance with regard to reduced progression free survival (PFS) and overall survival (OS) has previously been demonstrated [17, 18, 19, 20, 21]. In addition, we demonstrated that patients with a marked increase of DTCs after platinum-based chemotherapy showed a significantly reduced PFS [22].

Based on the studies mentioned above, there is increasing evidence that DTCs could reflect cancer progression. Thus, DTCs could be used as novel targets for additional therapeutic strategies. In this study, we analyzed whether our previously reported negative prognostic influence of DTCs with regard to reduced PFS and OS 1) was related to the persistence of DTCs after platinum based chemotherapy and/or 2) might have arisen from a cellular phenotype showing stem cell characteristics.

## RESULTS

### Detection of DTCs

Before therapy (BT), DTCs were detected in 33/79 patients (42%) with a median number of 4 DTCs (range 1–37). After therapy (AT), 32/79 patients (41%) were positive for DTCs (median cell number of 8 cells (range 1–100) (Table 1). DTCs were found in 13 patients BT and AT, in 20 patients only BT and in 19 patients only AT, respectively. DTCs were not detected in samples taken BT or AT from 27 patients (Table 2).

**Table 1:** Patient characteristics at the time of primary diagnosis

Total	79
Age	median 60 years, (26–86)
FIGO stage	
I–II	21 (26%)
III	48 (61%)
IV	10 (13%)
Nodal status	
N_o_	32 (40,5%)
N_1_	32 (40,5%)
N_x_	10 (19%)
Grading	
I–II	44 (56%)
III	33 (42%)
Unknown	2 (2%)
Residual tumor	
Macroscopic	
Complete resection	49 (62%)
Any residual tumor	30 (38%)
Histologic type	
Serous	47 (60%)
Mucinous	9 (11%)
Other	23 (29%)
DTC	
Before therapy	33 (42%)
After therapy	32 (41%)
Survival	
PFS^1^	median 15 months, (4–87 months)
OS^2^	median 62 months, (10–128 months)
Alive	44 (56%)
Dead	33 (42%)
Unknown	2 (2%)
Recurrence	
No relapse	25 (32%)
Relapse	53 (67%)
Unknown	1 (1%)
Platinum resistance	
Platinum sensitive	60 (76%)
Platinum resistant	19 (24%)

1 PFS: progression-free survival

2 OS: overall survival.

**Table 2:** Prognostic significance of DTCs before and after therapy with regard to PFS and OS

Status	Number of patients (*n*)	PFS (*p*-value)	OS (*p*-value)
Total	79		
DTC_pos_ before therapy	33	0.06	**0.02**
DTC_pos_ after therapy	32	0.35	0.98
DTC_pos_/DTC_pos_	13	0.06	0.07
DTC_neg_/DTC_neg_	27	0.77	0.31
DTC_pos_/DTC_neg_	20	0.46	0.25
DTC_neg_/DTC_pos_	19	**0.03**	0.18

### Prognostic significance of DTCs

**Figure 1:**
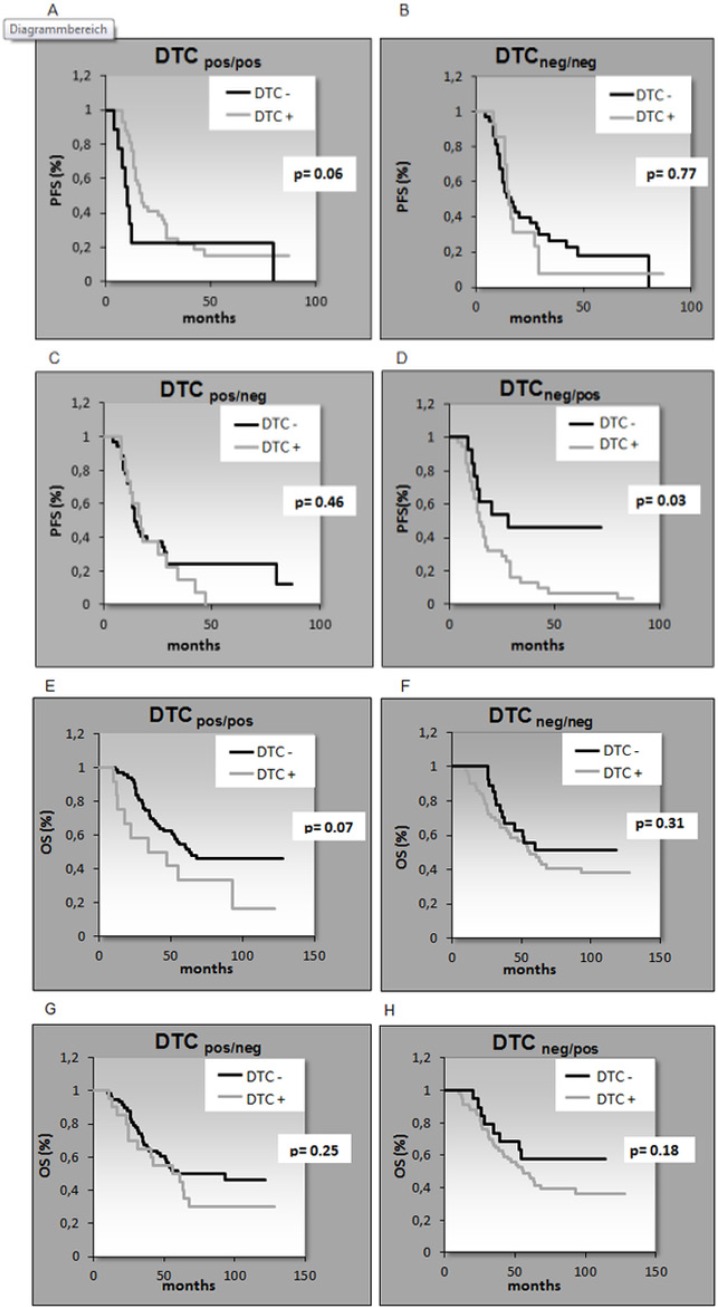
Kaplan-Meier analysis for the correlation of PFS (A–D) and OS (E–H) with DTC detection Patients initially DTC_neg_ before therapy but DTC_pos_ after therapy had a significant shorter PFS (*p* = 0.03) (Figure 1D). A. PFS DTC_pos/pos_, B. PFS DT_neg/neg_, C. PFS DTC_pos/neg_, D. PFS DTC_neg/pos_, E. OS DTC_pos/pos_, F. OS DTC_neg/neg_, G. OS DTC_pos/neg_, H. OS DTC_neg/pos_.

After a median follow up time of 62 months (range 10–128 months), 44 patients (56%) were still alive and 33 patients (42%) had died. The median follow-up time for PFS was 15 months (range 4–87 months) resulting in 53 (67%) relapses while 25 patients (32%) had no relapse (Table 1). The presence of DTCs BT significantly correlated with reduced OS (*p* = 0.02) and patients initially DTC_neg_ BT but DTC_pos_ AT had a significant shorter PFS (*p* = 0.03) (Table 2 and Figure 1). The persistence of DTCs resulted in a shorter PFS and OS reaching borderline significance (*p* = 0.06; *p* = 0.07).

### Evaluation of LIN28- and SOX-2-positive cells

**Figure 2:**
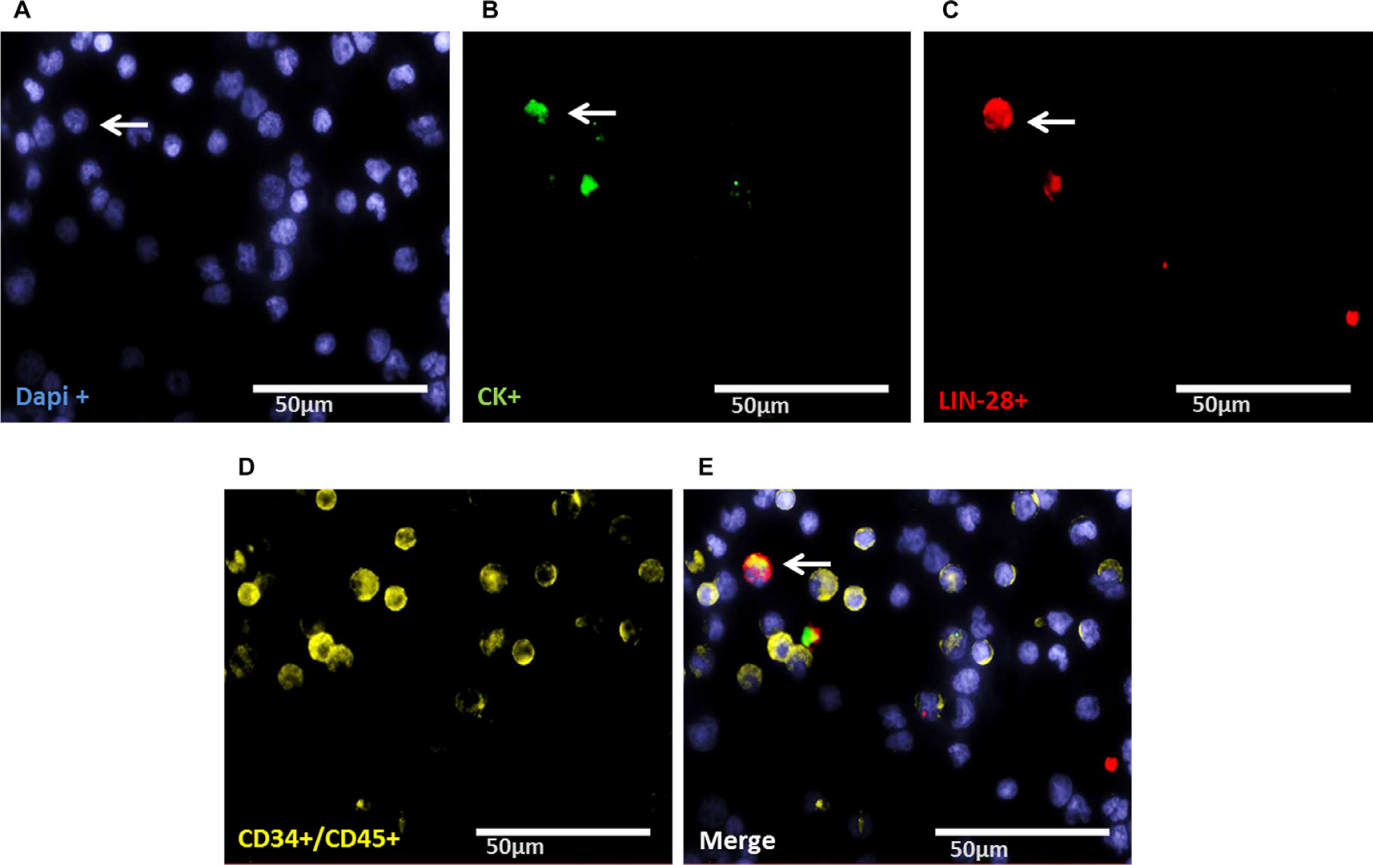
Representative four-fold immunofluorescence staining for CK_pos_/LIN-28_pos_ cells after therapy of patient No1 (**A**) Cell nuclei were stained with Dapi. (**B**) Indicates a CK_pos_ cell. (**C**) Alludes a cell with LIN-28_pos_ phenotype. (**D**) Shows CD34_pos_ and/or CD45_pos_ cells. (**E**) Indicates a merge of a DTC with the phenotype Dapi_pos_, CK_pos_, LIN-28_pos_, CD34_neg_ and CD45_neg,_ magnification at 63×.

**Figure 3:**
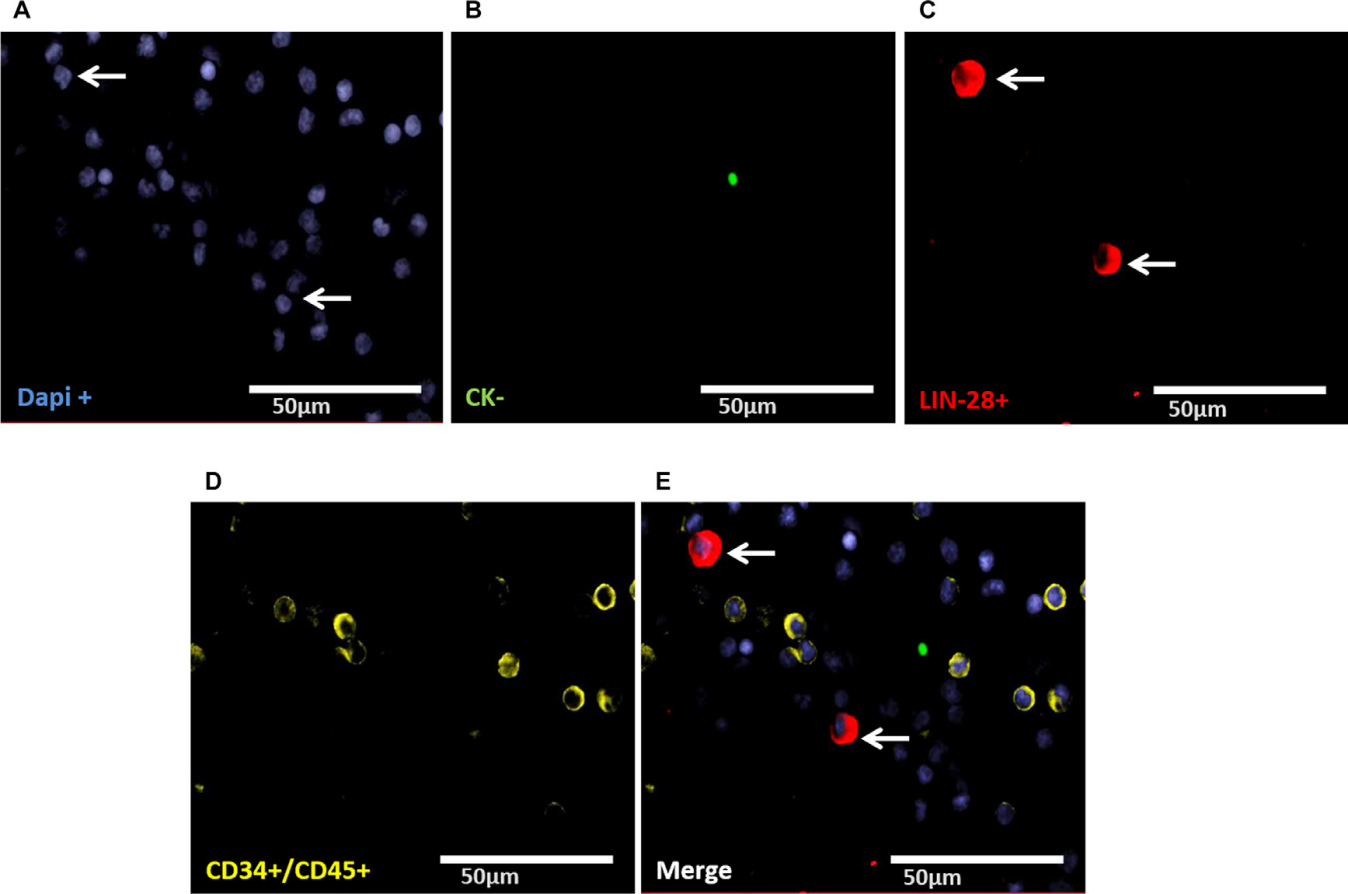
Representative four-fold immunofluorescence staining for CK_neg_/LIN-28_pos_ cells after therapy of patient No1 (**A**) Cell nuclei were stained with Dapi. (**B**) Indicates a CK_neg_ cell. (**C**) Alludes a cell with LIN-28_pos_ phenotype. (**D**) Shows CD34_pos_ and/or CD45_pos_ cells. (**E**) Indicates a merge of two DTCs with the phenotype Dapi_pos_, CK_neg,_ LIN-28_pos_, CD34_neg_ and/or CD45_neg,_ magnification at 63×.

**Figure 4:**
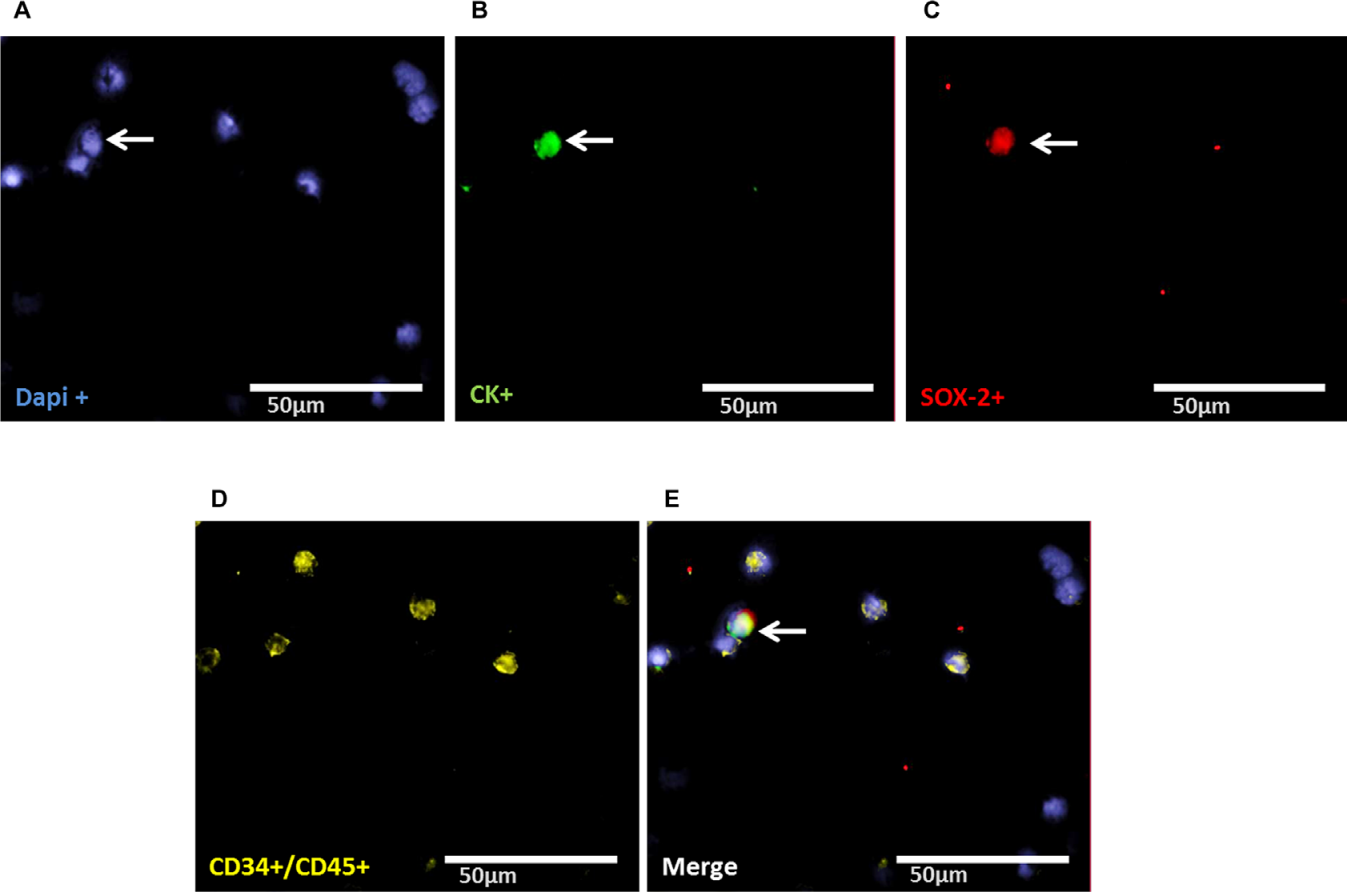
Representative four-fold immunofluorescence staining for CK_pos_/SOX-2_pos_ cells after therapy of patient No1 (**A**) Cell nuclei were stained with Dapi. (**B**) Indicates a CK_pos_ cell. (**C**) Alludes a cell with SOX-2_pos_ phenotype. (**D**) Shows CD34_pos_ and/or CD45_pos_ cells. (**E**) Indicates a merge of a DTC with the phenotype Dapi_pos_, CK_pos_, SOX-2_pos_, CD34_neg_ and/or CD45_neg,_ magnification at 63×.

**Figure 5:**
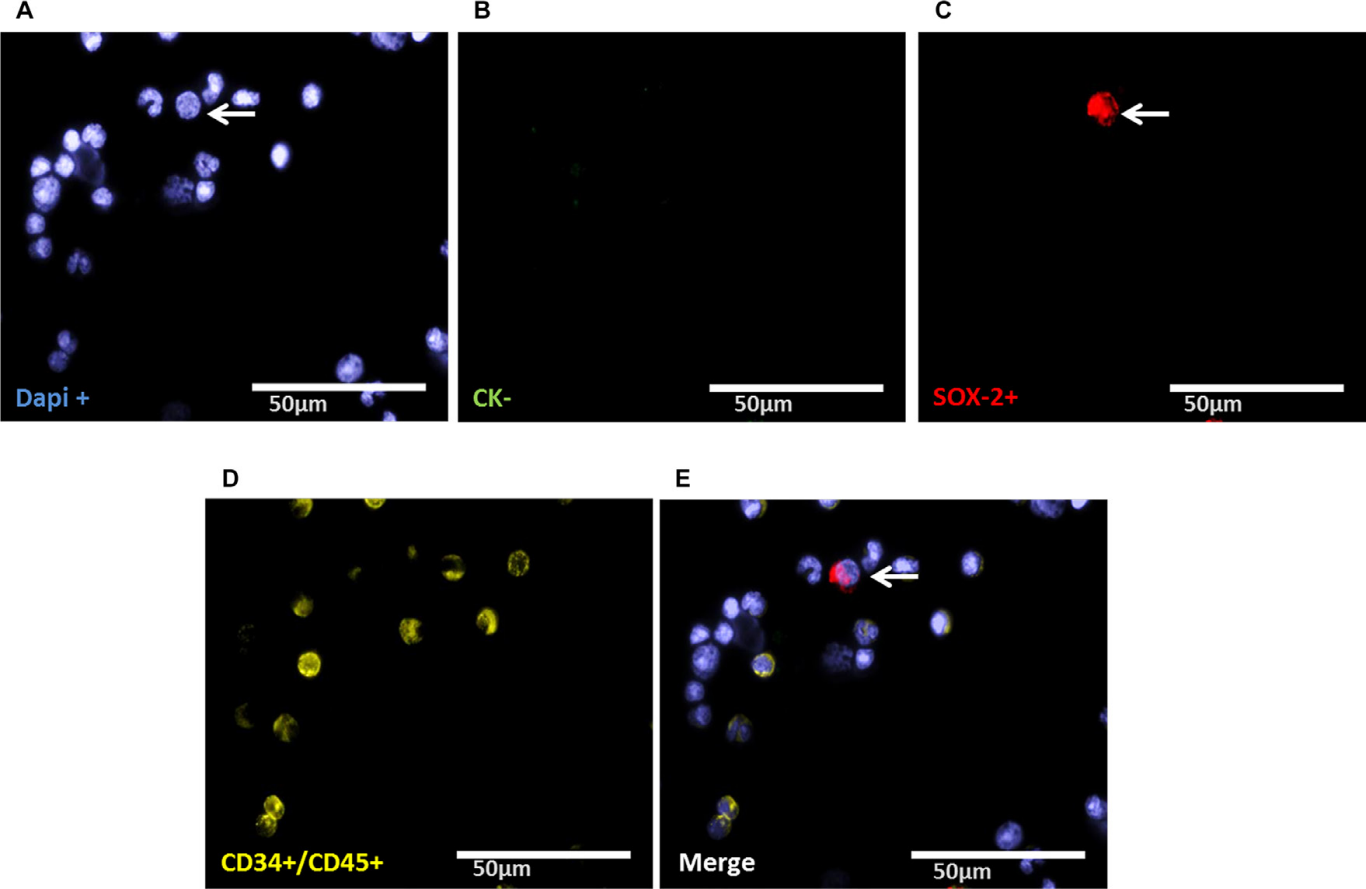
Representative four-fold immunofluorescence staining for CK_neg_/SOX-2_pos_ cells after therapy of patient No1 (**A**) Cell nuclei were stained with Dapi. (**B**) Indicates a CK_neg_ cell. (**C**) Alludes a cell with SOX-2_pos_ phenotype. (**D**) Shows CD34_pos_ and/or CD45_pos_ cells. (**E**) Indicates a merge of a DTC with the phenotype Dapi_pos_, CK_neg,_ SOX-2_pos_, CD34_neg_ and CD45_neg,_ magnification at 63×.

Staining of patient samples is shown in Figures 2–5. Controls are shown in Supplementary Figures S1–S3. A DTC was classified as a stem-like tumor cell if it had the following staining characteristics: Dapi_pos_, CD45_neg_, CD34_neg_, SOX-2_pos_/LIN-28_pos_ and CK_pos_ or CK_neg_ (Figures 2–5). The Kasumi cell line was used to establish CD34 expression (Supplementary Figure 1) and BM samples from healthy donor patients for CD34- and CD45-expression (Supplementary Figures 2 and 3).

### Detection of LIN-28- and SOX-2-positive cells

DTCs from 10 patients were analyzed BT and AT for SOX-2 and LIN-28 positive cells AT (Table 3; columns 1 and 2; Supplementary Table 1). 8/10 patients had at least five DTCs as detected by immunocytochemistry using A45/B-B3. In addition, 2/10 patients (patient 2 and 5) were DTC_neg_ AT but DTC_pos_BT. As apparent from Table 3, AT CK_pos_/LIN-28_pos_ cells were detected in 9/10 patients [median 2 cells (range 1–5)] and CK_neg_/LIN-28_pos_ cells in 7/10 patients [median 3 cells (range 1–11)], respectively. CK_pos_/SOX-2_pos_ cells were detected in 6/8 patients [median 2 cells (range-0–4)] and CK_neg_/SOX-2_pos_ cells were found in 7/8 patients [median 4 cells (range 1–11)]. Patients two and five, who were characterized as DTC_neg_ AT by immunocytochemistry but were positive BT (37 and 6 DTCs, respectively) were included in our analysis for stem cell-associated markers. Interestingly, these two patients harbored 1–2 LIN-28_pos_ and SOX-2_pos_ cells in their BM AT. Thus, we evaluated LIN-28-/SOX-2-positive cells BT in cases vice versa, DTC_neg_ BT but DTC_pos_ AT (patients 1, 3 and 4) as well as in patient number 6 with persistent DTCs. As shown in Table 3, in patients who switched initially DTC_neg_ before but becoming DTC_pos_ AT (patients 1, 3 and 4), in patients who switched from being DTC_pos_ before but becoming DTC_neg_ AT (patients 2 and 5) as well as in patient number 6 with persistent DTCs (patient 6) a few LIN-28 as well as SOX-2-positive cells were present in BM even BT.

**Table 3:** Distribution of DTCs and LIN-28-/SOX-2-positive cells before and after therapy

			before therapy				after therapy			
Patient	DTC_pos_ before therapy (A45-B/B3)	DTC_pos_ after therapy (AF45-B/B3)	CK_pos_/ LIN- 28_pos_	CK_neg_/ LIN- 28_pos_	CK_pos_/ SOX- 2_pos_	CK_neg_/ SOX- 2_pos_	CK_pos_/ LIN- 28_pos_	CK_neg_/ LIN- 28_pos_	CK_pos_/ SOX- 2_pos_	CK_neg_/ SOX- 2_pos_
1	0	14	25	5	24	2	4	0	3	4
2	37	0	2	0	4	1	0	1	2	0
3	0	11	8	2	5	1	2	0	0	1
4	0	15	2	1	1	1	2	1	nsa	nsa
5	6	0	1	1	2	1	1	0	2	1
6	28	18	nsa	nsa	2	3	3	7	4	11
7	0	5	nsa	nsa	nsa	nsa	1	3	0	2
8	0	100	nsa	nsa	nsa	nsa	5	9	1	2
9	1	35	nsa	nsa	nsa	nsa	2	1	nsa	nsa
10	0	10	nsa	nsa	nsa	nsa	3	11	2	9

nsa: no slides available.

## DISCUSSION

To the best of our knowledge, this is the first study showing that DTCs, present after platinum based chemotherapy in primary ovarian cancer patients show stem cell characteristics. Furthermore, although *p* values reached borderline significance, these cells might be associated with worse outcome which finally has to be proven in a bigger patient cohort.

The rate of DTC detection in primary ovarian cancer before the administration of platinum-based chemotherapy has been reported to be 20% to 60%, depending on the method of detection used. Furthermore, their presence has been associated with worse outcome [17, 18, 19, 20, 21, 22]. The lack of significant correlation between DTCs and clinical outcome reported by other investigators may be due to their use of different antibodies for detection of DTCs [23, 24].

In this study, DTCs were present in the BM AT in 41% of the patients which is in accordance with our earlier data which also demonstrated that DTCs, still present AT, were non-apoptotic and their marked increase was associated with a significantly reduced PFS [22]. These findings suggest that the BM seems may to be a temporary homing site for isolated tumor cells, where they can persist and potentially induce recurrence of the disease. Analyzing 79 patients BT and AT, we confirm the negative prognostic influence of DTC detection with regard to OS [20]. We observed persistent DTCs in 16% of the patients which was associated with a shorter PFS and OS, reaching borderline significance. Interestingly, 24% of the patients that were initially characterized as DTC_neg_ BT and converted to DTC_pos_ AT had a significantly shorter PFS. The negative prognostic influence of these cells could be in alliance with a currently discussed hypothesis that some DTCs may have cancer stem cell features and may be the active source of metastatic spread in primary tumors, in addition to resistance to various chemotherapeutic agents and radiotherapy [25]. Two studies have confirmed a putative stem cell phenotype among DTCs in breast cancer patients [26, 27]. Here, we demonstrate that presence of DTCs that persist AT express the stem cell markers Lin-28 and/or SOX-2. We show that stem cell-like cells were present before the administration of chemotherapy. These findings may explain the significantly shorter PFS of patients who changed from DTC_neg_ BT to DTC_pos_AT. In addition, patients characterized as DTC_neg_ AT also harbored some Lin-28 and/or SOX-2 positive cells in their BM which may be responsible for a worse outcome. Until now, tumor stem cells have only been analyzed in ovarian tumor tissue, but not in DTCs. In this regard, previous studies have shown that LIN-28, SOX-2 as well as OCT-4 play a major role in carcinogenesis [10, 11, 12]. Wang et al., reported that SOX-2 targets SRC Kinase, a non-receptor tyrosine kinase that increases cell migration, invasion and adhesion of serous ovarian carcinoma cells [13]. Inhibition of either LIN-28 or Oct-4 expression decreases cell viability. The combined repression of both LIN-28 and Oct-4 results in synergistic inhibition of cancer cell growth and survival of ovarian cancer cell lines [9]. Expression of SOX-2 has been investigated by immunohistochemistry analysis of normal ovarian epithelial, serous and mucinous cystadenoma and cystadenomacarcinoma specimens [28] and LIN-28 was overexpressed in different epithelial tumors including breast, lung, colon and ovarian cancer [29]. Furthermore, SOX-2 may be crucial for the development of chemotherapy resistance. Yang et al., analyzed SOX-2 expression in clinical tissue samples and ovarian cancer cell lines using immunohistochemistry and real-time PCR and demonstrated that SOX-2 was overexpressed in paclitaxel-resistant cells [30]. In ovarian cancer patients receiving taxanes, expression of SOX-2 was shown to be correlated with chemotherapy resistance and a shorter PFS whereas patients receiving non-taxane based chemotherapy showed no significant response influence [31]. Since the patients in our study have also received a combined therapy with paclitaxel and carboplatin, it may be possible that SOX-2 expression on DTCs was associated with chemotherapy resistance. However, mechanisms associated with chemotherapy resistance in ovarian cancer still remain unclear. Functionally, primary platinum-resistance, defined as platinum-free treatment interval of less than 6 months observed in up to 20% of ovarian cancer patients, can be the result of either increased tolerance towards DNA-platinum-adducts or enhanced DNA-repair capacity of tumor cells [32, 33, 34]. In this context, we recently demonstrated that ERCC1_pos_ (excision-repair cross-complementing rodent repair deficiency, complementation group 1 nuclease) circulating tumor cells (CTCs) constituted an independent predictor, not only for OS but also for PFS in our ovarian cancer patients. Most interestingly, the presence of ERCC1_pos_ CTCs at primary diagnosis was an independent predictor for platinum-resistance whereas ERCC1-expression in the corresponding primary tumor tissue predicted neither platinum-resistance, nor prognosis [35]. Consequently, assuming that CTCs must be spread into the circulation from existing pools in secondary organs, e.g. the BM, one might speculate that ERCC1_pos_ DTCs also exist and contribute to platinum resistance. Interestingly, DTCs present BT in our patients significantly correlated with clinical platinum resistance (data not shown).

In this study, we also detected CK_pos_/SOX-2_pos_ (LIN-28_pos_) as well as CK_neg_/SOX-2_pos_ (LIN-28_pos_) cells in all patients. We assume that two different cell types with expression of stem cell associated proteins may have been detected. It has been described that tumor cells undergo phenotypic changes, known as epithelial-mesenchymal transition (EMT), which allow them to migrate to sites of metastasis without being eliminated by conventional treatment [36]. Thus, CK_neg_/SOX-2_pos_ (Lin-28_pos_) cells might result from EMT while the CK_pos_/SOX-2_pos_ (LIN-28_pos_) epithelial phenotype may have remained unchanged.

Taking all these considerations into account, additional therapeutic strategies will be required to target signaling pathways concerning CSC. These studies will include mTOR inhibitors, acting downstream of the PI3K/AKT pathway [37], salinomycin [38] or a new synthetic curcumin analogue against ALDH1 and GSK-3ß [39]. Finally, approaching the tumor microenvironment, such as interrupting the immune cells and cytokines (e.g. IL-6, IL-8) as well as the immune checkpoints (PD1/PDL1) may provide additional new tools for immunological killing of cancer stem cells [40, 41, 42]

### Conclusion and limitation of the study

The cohort of our patients is probably too small to draw the final conclusion that a significant selection of stem cell marker-positive DTCs occurs during chemotherapy. Consequently, the results presented here should be viewed as a “proof of principle”, that DTCS with stem cell characteristics exist among DTCs that are present BT and persist AT. To the best of our knowledge, we are the only group that has a collection of BM cells harvested from primary ovarian cancer patients AT. In this regard, 79 paired samples from patients who consented to allow collection of their BM under local anesthesia AT for research purposes is a unique collection and would be difficult to achieve high patient numbers. Furthermore, based on the number of residual slides and methodological requirements, we only analyzed two stem cell markers. Ongoing studies will include other stem cell markers, such as OCT4 as well as resistance marker to finally elucidate the prognostic relevance of these cells.

## PATIENTS AND METHODS

### Patient characteristics

79 patients with primary ovarian cancer who presented at the Department of Gynecology and Obstetrics, University Hospital Essen between February 2004 and January 2010 were included in this analysis. Patient characteristics are documented in Table 1. The mean age was 60 years (range 26–86 years), the median follow-up time was 62 months (10–128 months) for OS and 15 months (4–87 months) for PFS. Written Informed consent was obtained from all patients and the study was approved by the Local Ethics Committee (05–2870). Tumors were classified according to the WHO classification of tumors of the female genital tract. Grading was conducted using the grading system proposed by Silverberg [43] and tumor staging was classified according to the Fédération Internationale de Gynécology et d'Obstétrique (FIGO 2009). The entire study population underwent primary radical surgery. Total abdominal hysterectomy, bilateral salpingo-oophorectomy, infragastric omentectomy, peritoneal stripping was performed and in addition to pelvic and para-aortic lymphadenectomy, if macroscopic complete resection was achieved. The most important aim of surgery was to achieve macroscopic complete tumor resection. Radical pelvic and para-aortic lymphadenectomy were only performed if complete tumor resection was achieved intraperitoneally following actual guidelines (www.ago-ovar.de). All patients received at least six cycles of carboplatinum AUC 5 and paclitaxel 175 mg/m2. Tumors were clinically defined as platinum-resistant if they recurred within six months after the completion of platinum-based chemotherapy.

### Cell lines

The human ovarian cancer cell line OVCAR-3 and the Kasumi-1 cell line were purchased from the American Type Culture Collection (ATCC, Manassas, VA, USA) and cultured in RPMI 1640 containing 10% (20% for Kasumi-1) fetal calf serum and 1% (100 U/ml) Penicillin-Streptomycin (Gibco™ by Thermo Fisher Scientific, Waltham MA, US). Cells were grown at 37°C in a humidified atmosphere with 5% CO2.

### Detection of DTCs

Between 10 and 20 ml BM were aspirated from the anterior iliac crests and processed within 24 hours. DTC selection and detection was performed based on the recommendations for standardized tumor cell detection [44]. Details of the staining procedure and cell detection have been described elsewhere [22]. Briefly, BM cells were isolated from heparinized BM (5000 U/ml BM) by Ficoll-Hypaque density gradient centrifugation (density 1.077 g/mol; Pharmacia, Freiburg, Germany) at 400 × g for 30 min. Slides were analyzed for DTCs by immunocytochemistry using the pan-cytokeratin antibody A45-B/B3. Microscopic evaluation of the slides (1 × 10^6^ mononuclear cells per slide) was carried out using the ARIOL system (Applied Imaging) according to the ISHAGE evaluation criteria [45].

### Detection of LIN-28- and SOX-2-positive cells

LIN-28- and SOX-2 positive cells were analyzed separately on additional slides of the same patients harboring at least five DTCs as detected by immunocytochemistry using the A45B-B3. Four-fold immunofluorescence staining was established using the OVCAR-3 cell line spiked into blood of healthy donors. Since CD34-positive normal hematopoietic stem cells comprise 1.5% of marrow mononuclear cells [46], we included CD34 in our analysis to exclude false-positive results. CD34 was analysed using the Kasumi-1 cell line since BM of healthy donors was difficult to obtain and only available from one donor (Supplementary Figure 1).

Slides were fixed with 4% Paraformaldehyde for 10 min, permeabilized with 0.1% Triton-X-100 for 15 min and subsequently washed with TBS and Triton-X-100 three times for five min. Slides were stained with SOX-2 (Anti-human SOX-2, 1:50, R & D Systems, USA), LIN-28 (LIN-28, 1:350, Rabbit polyclonal ab46020, Abcam, UK), C11 (anti-PAN-Cytokeratin, 1:400, FITC-labelled, GeneTex, USA), CD34 (Alexa Flour anti-human 647, 1:100, Cy5 labelled, Biolegend, USA) and CD45 (Alexa Fluor 647;sc1178 Santa Cruz, USA) incubated in a wet chamber for one hour at room temperature. Subsequently, slides were incubated with DAPI [pre diluted in Phosphate Buffered Saline (1:250) and further diluted in AB diluent (1:20, Dako, Germany)], and TRITC-labelled donkey anti-goat 594 (SOX-2), donkey anti-rabbit 594 (LIN-28) (both1:100, Invitrogen, USA) under the same conditions followed by three washing steps for five minutes. Moreover, negative controls for primary antibodies were prepared by staining the spiked OVCAR-3 cell line with the secondary antibodies for 30 minutes under the same conditions. The slides were mounted with Dako fluorescent mounting medium s3023 and a coverslip and dried overnight in a cooling chamber. Counting was performed visually, using an immunofluorescence microscope (Axioplan 2 Imaging Zeiss Germany, Metasystems) and Isis Fish imaging system V5.3 (Meta Systems, Germany) at a magnification of 40× or 63×.

### Statistical analysis

Survival analysis was performed by using Winstat (2012.1) an upgrade of Microsoft Excel. Survival intervals were screened from the time of BM aspiration at first diagnosis to the time of death or first time of relapse, defined as either local recurrence or distant metastasis. Kaplan-Meier curves were established using the log-rank test to evaluate univariate significance of the parameters.

## SUPPLEMENTARY MATERIALS FIGURES AND TABLES


