# Supplementary material for: Analysis of disseminated tumor cells before and after platinum based chemotherapy in primary ovarian cancer. Do stem cell like cells predict prognosis?
Source: Oncotarget. 2016 Apr 1;7(18):26454–64. doi: 10.18632/oncotarget.8524 (PMC5041992; doi:10.18632/oncotarget.8524)
Supplement: Supplementary file 1 [file oncotarget-07-26454-s001.pdf]

Analysis of disseminated tumor cells before and after platinum based chemotherapy in primary ovarian cancer. Do stem cell like cells predict prognosis?

Supplementary Materials

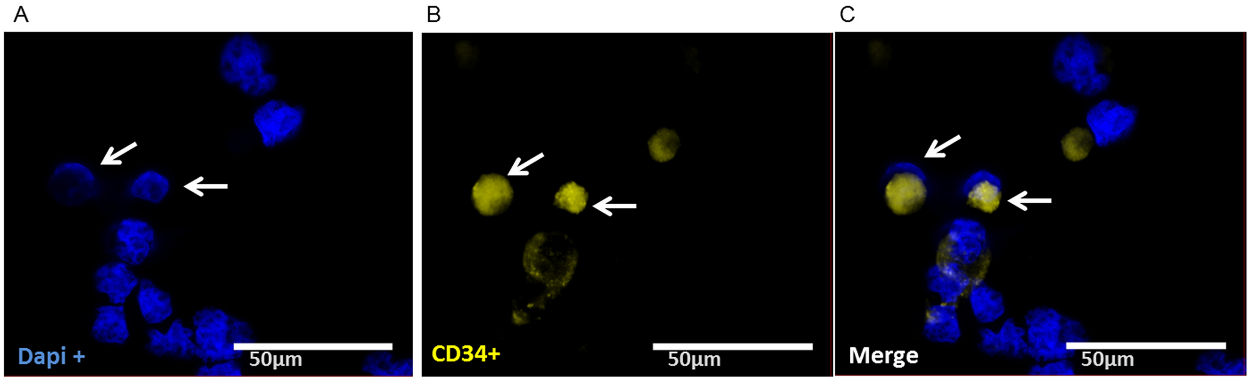

Supplementary Figure S1: Representative staining of immunofluorescence staining of the kasumi-1 cell line used as positive control for the detection of hematopoietic stem cells (CD34$_{pos}$). (**A**) Cell nuclei were stained with Dapi. (**B**) The arrows show two CD34$_{pos}$ cells. (**C**) Indicates a merge of Dapi$_{pos}$ and CD34$_{pos}$ cells, magnification at 63×.

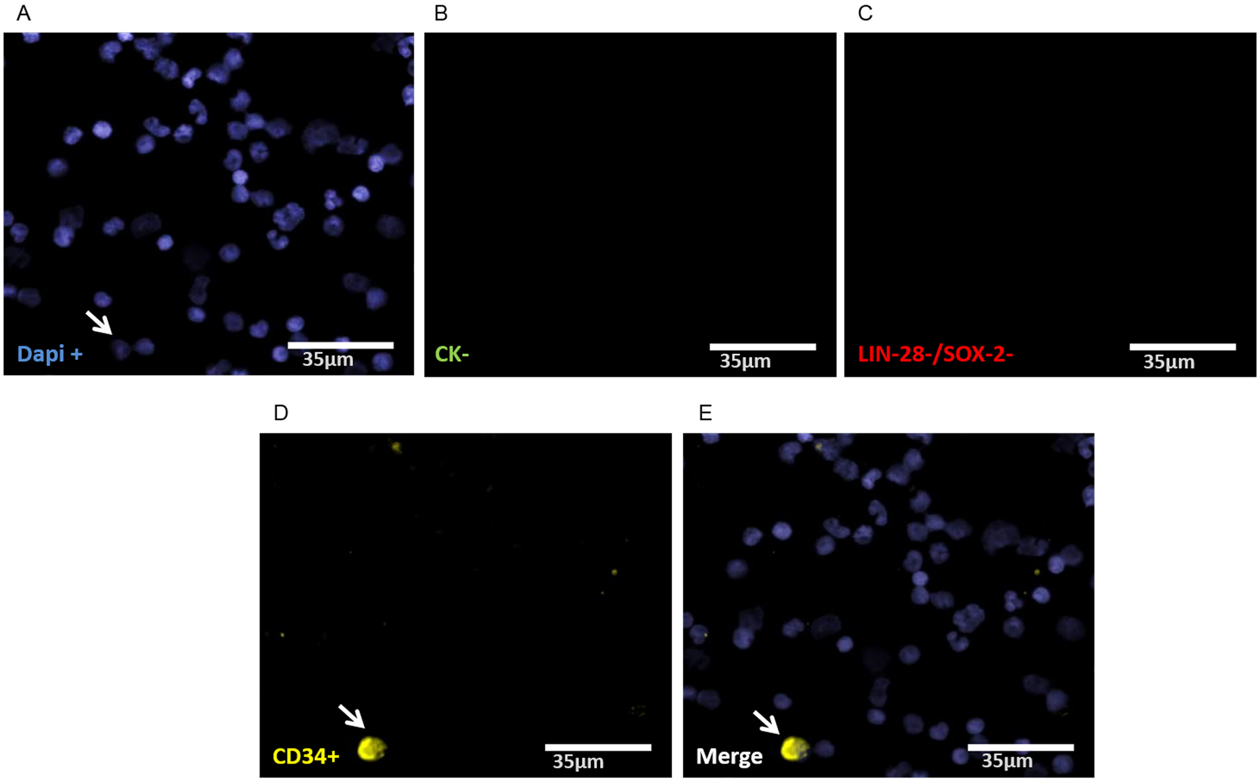

Supplementary Figure S2: Representative four-fold immunofluorescence staining for detection of hematopoietic stem cells (CD34$_{pos}$) in the bone marrow of a healthy donor using primary and secondary antibody for LIN-28 and SOX-2. (**A**) Cell nuclei were stained with Dapi. (**B**) Indicates CK$_{neg}$ cells. (**C**) Alludes a cell with LIN-28$_{neg}$/SOX-2$_{neg}$ phenotype. (**D**) Shows CD34$_{pos}$ cell. (**E**) Indicates a merge of a hematopoietic stem cell with Dapi$_{pos}$, CK$_{neg}$, SOX-2$_{neg}$/LIN-28$_{neg}$, CD34$_{pos}$ phenotype, magnification at 40×.

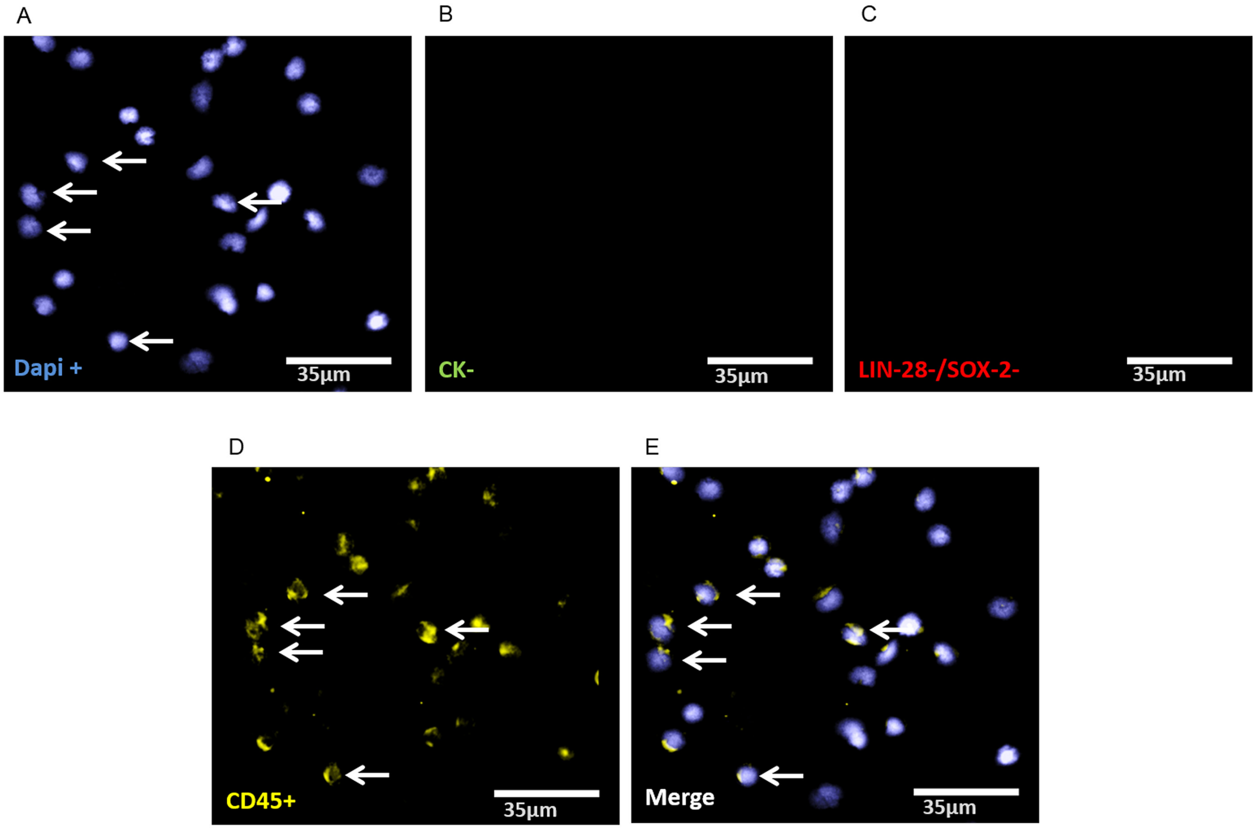

Supplementary Figure S3: Representative four-fold immunofluorescence staining for detection of leukocytes ($CD45_{pos}$) in the bone marrow of a healthy donor using primary and secondary antibody for LIN-28 and SOX-2. (**A**) Cell nuclei were stained with Dapi. (**B**) Indicates CK_{neg} cells. (**C**) Alludes a cell with $LIN\text{-}28_{neg}$/$SOX\text{-}2_{neg}$ phenotype. (**D**) Shows a $CD34_{pos}$ cell. (**E**) Indicates a merge of hematopoietic stem cells with $Dapi_{pos}$, CK_{neg}, $SOX\text{-}2_{neg}$/$LIN\text{-}28_{neg}$, $CD45_{pos}$ phenotype, magnification at 40×.

Supplementary Table S1: Characteristics of patients analyzed for DTCs with stem cell character

Total	10
Age	median 58 years, (30–77)
FIGO stage	
I–II	1 (10%)
III	6 (60%)
IV	3 (30%)
Nodal status	
N_o	3 (30%)
N_1	2 (20%)
N_x	5 (50%)
Grading	
I–II	7 (70%)
III	3 (30%)
Residual tumor	
Macroscopic	
Complete resection	4 (40%)
Any residual tumor	6 (60%)
Histologic type	
Serous	8 (80%)
Other	2 (20%)
Survival	
PFS[1]	median 18 months, (4–72 months)
OS[2]	median 53 months, (13–101 months)
Alive	4 (40%)
Dead	6 (60%)
Recurrence	
No relapse	2 (20%)
Relapse	8 (80%)

[1]PFS: progression-free survival, [2]OS: overall survival.
